# Diagnosis of fungal opportunistic infections in people living with HIV from Guatemala and El Salvador

**DOI:** 10.1111/myc.13368

**Published:** 2021-09-29

**Authors:** Diana Forno, Blanca Samayoa, Narda Medina, Eduardo Arathoon, Carlos Rodolfo Mejia, Remei Gordillo, Rolando Cedillos, Jose Rodas, Angela Ahlquist Cleveland, Tom Chiller, Diego H. Caceres

**Affiliations:** ^1^ Division of Global HIV & TB at the Central America Regional Office for the Centers for Disease Control and Prevention (CDC) Guatemala City Guatemala; ^2^ Asociación de Salud Integral Guatemala City Guatemala; ^3^ Facultad de Ciencias Químicas y Farmacia Universidad de San Carlos de Guatemala Guatemala City Guatemala; ^4^ Clinica Familiar “Luis Ángel García” Hospital General San Juan de Dios Guatemala City Guatemala; ^5^ Hospital Roosevelt Guatemala City Guatemala; ^6^ Hospital Nacional Rosales San Salvador El Salvador; ^7^ Mycotic Diseases Branch CDC Atlanta GA USA; ^8^ Department of Medical Microbiology Radboud University Medical Center and Center of Expertise in Mycology Radboudumc/CWZ Nijmegen The Netherlands

**Keywords:** cryptococcosis, diagnosis, histoplasmosis, HIV, opportunistic infections, rapid tests

## Abstract

**Objectives:**

Histoplasmosis and cryptococcosis are important public health problems in people living with HIV (PLHIV) in Central America. Conventional laboratory tests, such as culture and microscopy, are not optimal; however, antigen (Ag) tests are rapid, highly sensitive, and specific for diagnosis of fungal opportunistic infections (OI). The aim of this study was to describe the results of a laboratory‐based surveillance system for histoplasmosis and cryptococcosis.

**Methods:**

An observational cross‐sectional study based on laboratory surveillance, was carried out in two hospitals in Guatemala and one hospital in El Salvador, between July 2012 and December 2014. Diagnosis of histoplasmosis and cryptococcosis in PLHIV were performed by culture and Ag test.

**Results:**

A total of 160 PLHIV were diagnosed with fungal OI, of which, 96 (60%) were diagnosed with histoplasmosis, 62 (39%) were with cryptococcosis, and two patients (1%) were diagnosed with both fungal diseases. Of the 160 patients analysed in this study, 94 (59%) were diagnosed using only an Ag assay. CD4 cell count data were available for 136 (85%) patients; 127 (93%) patients had a CD4 count <200; and 90 (66%) had counts <50 CD4 cells per µl. Antiretroviral therapy utilisation at diagnosis was low (33%). Seventy‐one out of 160 (44%) were co‐infected with tuberculosis or other OIs.

**Conclusion:**

More than half of the patients in this study were diagnosed only by rapid laboratory Ag tests. A high per cent of the patients had advanced HIV disease.

## 1 INTRODUCTION

Fungal pathogens commonly found in Central America include *Histoplasma capsulatum* and *Cryptococcus neoformans/gattii*. People at high risk for these fungal opportunistic infections (OI) include individuals at the extremes of age, people undergoing immunosuppressive therapy, solid organ transplant recipients and people living with HIV (PLHIV) with advance HIV disease.^1^, ^2^ Despite advances in the treatment of PLHIV, the incidence and mortality associated with both diseases in PLHIV remain high (~30%).^3^, ^4^ Diseases caused by *H. capsulatum* and *C. neoformans/gattii* are common and important public health problems in PLHIV in Central and South American countries, and mortality rates vary from 18% to 48%.^3^, ^4^, ^5^

In 2019, there were 120,000 new HIV infections in Latin America, with 34,416 cases reported in Guatemala and the El Salvador representing nearly one third of the burden of HIV in the region.^6^ High mortality is seen among people who develop progressive disseminated histoplasmosis. Data from a cohort of HIV/AIDS patients with suspected histoplasmosis in Guatemala suggest that mortality from patients may have been as high as 44% before 2009, when technology of antigen (Ag) testing became available in the country.^7^ Over 90% of the meningitis cases caused by *C. neoformans*, known as cryptococcal meningitis, occur in PLHIV, a condition that represents the main risk factor for the development of this mycosis. Cryptococcal meningitis is the most important clinical presentation in these patients and is associated with high mortality rates, from 9% to 70% depending on the region of the world.^8^, ^9^

Conventional laboratory tests to diagnose histoplasmosis, such as culture, are routine recommended per national guidelines, but are not optimal. Culture requires a long incubation (up to 4 weeks), and microscopy requires obtaining invasive samples and has low sensitivity (50%).^1^ In cryptococcosis, India ink microscopy and culture have variable sensitivity (50%–80% and 64%–90%, respectively), and require specific laboratory training.^2^ The Centers for Disease Control and Prevention (CDC) developed an in‐house enzyme‐linked immunosorbent assay to detect *H. capsulatum* antigenuria (*Hc* ELISA) and diagnose histoplasmosis. This test was validated in Guatemala and Colombia and showed high sensitivity and specificity in both countries.^10^, ^11^ Ag detection tests have a sensitivity above 90% for the diagnosis of cryptococcosis. Additionally, Ag tests are relatively inexpensive, easy to perform and can be completed in <30 min using basic supplies. Currently, IMMY^®^ (Immuno‐Mycologics) developed a point‐of‐care cryptococcal Ag lateral flow assay test (CrAg^®^LFA) for early and rapid detection of cryptococcal Ag in serum and cerebral spinal fluid (CSF) for cryptococcosis diagnosis.^12^

The burden of fungal infections, including histoplasmosis and cryptococcosis, is underreported in Central America, and there was no data available at the time of the study although recent studies show that these fungal infections are relevant problems in PLHIV. A study done in Panama, Honduras and Nicaragua in hospitalised PLHIV demonstrated that two out of ten patients tested with a *Histoplasma* Ag detection ELISA, and one out of ten patients tested with the *Cryptococcus* Ag LFA tested positive.^13^ Another study from Guatemala revealed an incidence of 16% (*n* = 317) for OI (tuberculosis [TB], non‐TB mycobacteria infections, histoplasmosis and cryptococcosis) in PLHIV. This publication also described that both infections, histoplasmosis and cryptococcosis, affected two‐thirds (188 of 317) of PLHIV with OI.^9^ Presently, there is no data available on the prevalence or incidence of these OI from El Salvador.

Both CDC *Hc* ELISA and CrAg^®^LFA provide results much faster than other available laboratory tests. The aim of this study was to describe the results of a laboratory‐based surveillance system for histoplasmosis and cryptococcosis in countries from Central America.

## 2 MATERIALS AND METHODS

### 2.1 Study design

An observational, cross‐sectional study focused on the diagnosis of histoplasmosis and cryptococcosis in PLHIV. This laboratory‐based surveillance was carried out in two hospitals in Guatemala and one hospital in El Salvador, between July 2012 and December 2014. The clinical diagnosis or case definition of cryptococcosis and histoplasmosis was based on laboratory results, following international recommendations for the diagnosis of invasive fungal infections.^14^ All patients were treated according to local guidelines for fungal infections.

### 2.2 Laboratory testing

Diagnosis of histoplasmosis and cryptococcosis was performed by culture from any of the following samples: blood, tissue, sterile fluids or respiratory specimens. The CrAg^®^LFA and *Hc* ELISA were also used to diagnose cryptococcosis and histoplasmosis, respectively, these assays were performed following manufacturers recommendations.^10^, ^15^

Before enrolment and testing of patients, all personnel involved in this surveillance trained in assay performance and data collection. Samples were prospectively collected, and testing was performed in site.

### 2.3 Data collection and statistical analysis

A standardised data collection form was developed in Microsoft Access consisting of variables for clinical data, laboratory testing and outcome of patients. Absolute and relative frequencies were calculated to describe the demographic, clinical and laboratory characteristic of PLHIV with histoplasmosis and cryptococcosis. To identify differences in means or medians, we used the Student's *t* test or Mann–Whitney *U* test, as appropriate. For differences in proportions, we used chi‐squared test. A *p* value <0.05 was considered statistically significant. All analyses were performed using the software STATA 11 (StataCorp. 2009. Stata Statistical Software: Release 11: StataCorp LP) and EPIDAT 3.1.

### 2.4 Ethical considerations

This study was reviewed in accordance with the US CDC human research protection procedures and was determined to be non‐research. Local approvals were provided by clinical research ethics committee from Clínica Familiar Luis Angel Garcia and Hospital Roosevelt in Guatemala City, Guatemala, and Hospital Nacional Rosales, San Salvador, El Salvador.

## 3 RESULTS

**FIGURE 1:**
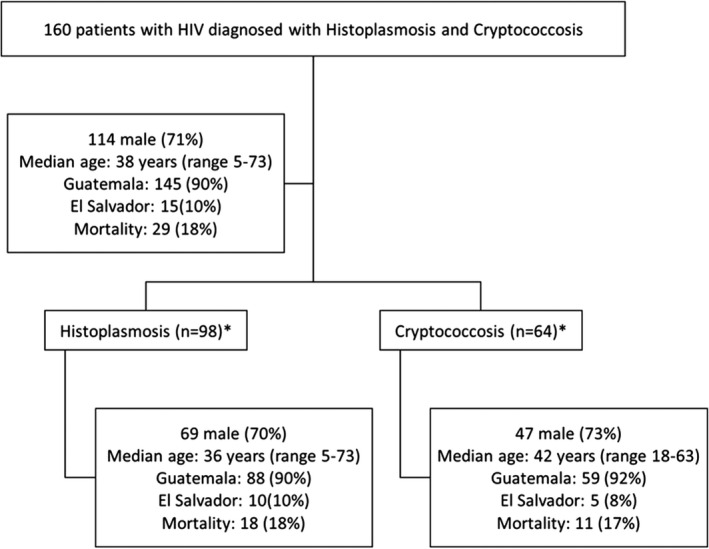
Demographic information for HIV patients with fungal opportunistic infections. *Two patients were diagnosed with co‐infection of histoplasmosis and cryptococcosis

We identified 160 PLHIV who were diagnosed with histoplasmosis or cryptococcosis based on positive culture or rapid test results. A total of 75 (47%) PLHIV in our cohort had been diagnosed with HIV within 3 months of the diagnosis of fungal OI. The median age was 38 years (range: 5–73) and 114 (71%) were male. At the time of diagnosis, 88 (55%) were hospitalised. Most patients were from Guatemala (*n* = 145, 90%), 14 (9%) were from El Salvador, and one (1%) was from Honduras but lived in El Salvador at time of diagnosis. Ninety‐six (60%) patients met the criteria as a case of histoplasmosis, 62 (39%) for cryptococcosis and two patients (1%) were diagnosed with both fungal diseases (Figure 1; Table 1). The overall 30‐day mortality was 18% (29/160), with mortality in patients with histoplasmosis being 18% (18/98) and that in patients with cryptococcosis being 17% (11/64). There were no statistically significant differences in mortality between patients with histoplasmosis or cryptococcosis (*p* = 0.87; Figure 1).

**TABLE 1:** Characteristics of patients with fungal opportunistic infections

Patient characteristics	Total (*N* = 160)	Histoplasmosis (*N* = 98)	Cryptococcosis (*N* = 64)	*p*
	*n* (%)	*n* (%)	*n* (%)	
Laboratory results				
Ag test only	94 (59)	65 (67)	29 (45)	0.012*
Ag test and culture	42 (26)	20 (20)	24 (38)	0.017*
Culture only	24 (15)	13 (13)	11 (17)	0.781
HIV infection status Total cases with CD4 count data available				
CD4 count, overall median (range)^a^	27 (1–927)	29 (1–430)	25 (2–927)	0.825
<50 cells per µl	90 (66)	58 (67)	33 (65)	0.961
50–100 cells per µl	24 (18)	13 (15)	12 (24)	0.300
101–200 cells per µl	13 (10)	8 (9)	5 (10)	0.854
>200 cells per µl	9 (7)	8 (9)	1 (2)	0.192
On ARV therapy at diagnosis	50 (31)	36 (37)^b^	16 (25)^b^	0.403
Clinical manifestations, treatment, and outcomes				
Respiratory symptoms	66 (41)	51 (52)	15 (23)	<0.001*
Gastrointestinal symptoms	74 (46)	59 (60)	15 (23)	<0.001*
Skin/mucosal lesions	21 (13)	15 (15)	6 (9)	0.391
Neurologic symptoms	67 (42)	17 (17)	50 (78)	<0.001*
Co‐infections				
Any co‐infections	71 (44)	43 (44)	31 (48)	0.683
Tuberculosis	33 (21)	18 (18)	16 (25)	0.414
Other infections	36 (23)	23 (23)	13 (21)	0.719
Histoplasmosis/cryptococcosis	2 (1)	2 (2)	2 (3)	0.933
Mortality within 30 days of diagnosis	29 (18)	18 (18)	11 (17)^c^	0.985
30 days mortality based on Ag test				
Positive result	—	12/85 (14)	8/38 (21)	—
Negative result	—	0/5 (0)	0/2 (0)	—
No done	—	6/8 (75)	3/16 (19)	—
30 days survival based on treatment				
Treated	102/117 (87)	71/80 (89)	33/39 (85)	—
Untreated	29/43 (67)	9/18 (50)	20/25 (80)	—

Abbreviation: Ag, antigen.

^a^ Data from 136 patients: 85 with histoplasmosis, 49 with cryptococcosis and 2 with histoplasmosis and cryptococcosis.

^b^ Two patients co‐infected with histoplasmosis and cryptococcosis.

^c^ Eight patients were diagnosed with meningitis.

* *p* < .05.

Of the 160 patients with histoplasmosis and cryptococcosis, 94 (59%) were diagnosed by Ag test alone, 42 (26%) were diagnosed by both Ag test and fungal culture, and 24 patients (15%) were diagnosed by fungal culture alone. Eighty‐four of the 98 (86%) cases of histoplasmosis were diagnosed using Ag test. For patients with cryptococcosis, 53 of 64 (83%) were diagnosed by detection of *Cryptococcus* Ag (24 of them with simultaneous positive culture). Of those PLHIV with cryptococcosis, 37 (58%) had a positive Ag test in CSF (Table 1). Five additional patients had positive *Cryptococcus* culture in CSF, having a total of 39 of the 64 cryptococcosis cases with cryptococcal meningitis (61%).

All patients showed the typical clinical symptoms of both fungal infections. At time of diagnosis, gastrointestinal manifestations were the most frequent clinical symptom identified in 74 of the 160 patients (46%) and was more frequent in patients with histoplasmosis (*n* = 59; 60%) than patients with cryptococcosis (*n* = 15; 23%; *p* < 0.001). Sixty‐six of 160 (41%) patients presented with respiratory symptoms, most commonly in patients with histoplasmosis (*n* = 51; 52%) compared with the cryptococcosis patients (*n* = 15; 23%; *p* < 0.001). Skin and mucosal lesions were present in 21 of 160 (13%) patients; 15 patients with histoplasmosis (*n* = 15, 15%) and in 6 patients with cryptococcosis (*n* = 6, 9%; *p* = 0.391). Finally, neurologic symptoms were present in 67 of the 160 patients and were more frequent in patients with cryptococcosis (*n* = 50; 78%) compared to patients with histoplasmosis (*n* = 17; 17%; *p* < 0.001; Table 1).

CD4 cell count data were available for 136 (85%) patients; 127/136 (93%) patients had a CD4 count <200, and 90/136 (66%) had counts <50 CD4 cells per µl. Among patients with histoplasmosis, 58/87 (67%) presented with counts <50 CD4 cells per µl. Among patients with cryptococcosis, 33/51 (65%) presented with counts <50 CD4 cells per µl (Table 1).

The most common risk factors associated with fungal OI were dirt removal (17%) and exposure to bird faeces (14%), with no statistically significant difference between risk for both types of fungal OI. Seventy‐one out of the 160 (44%) patients presented with co‐infections, TB was most common (*n* = 33; 21%; Table 1).

**FIGURE 2:**
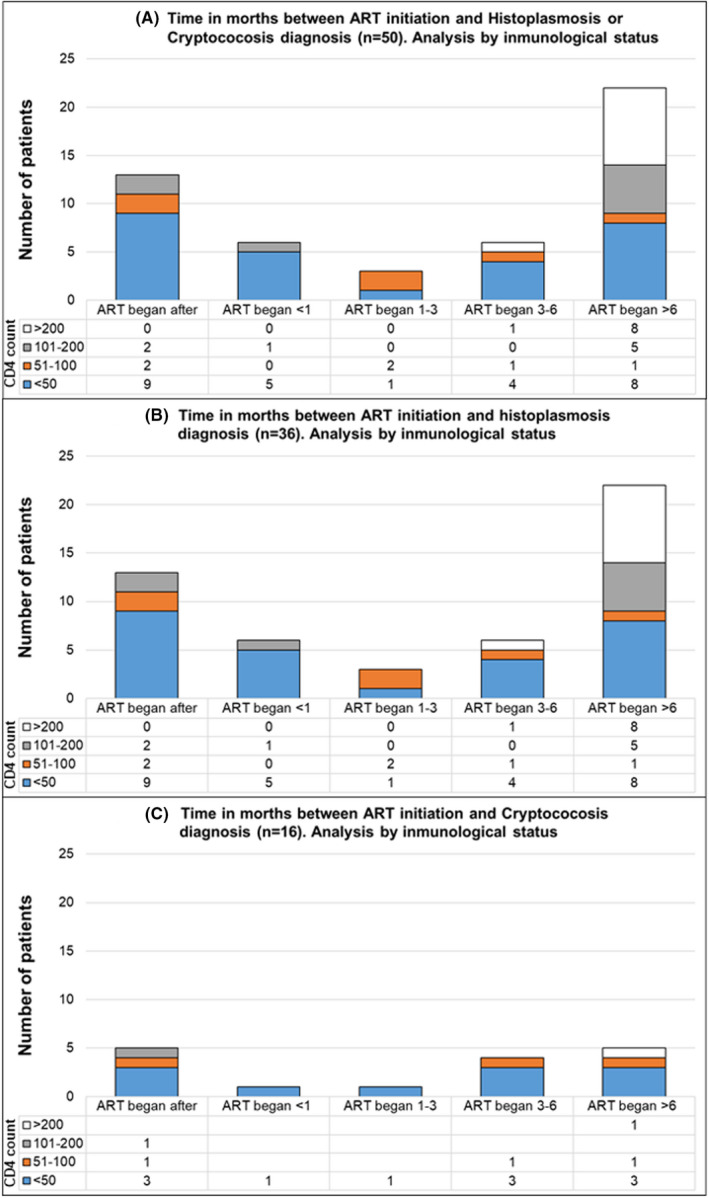
Time between antiretroviral therapy (ART) initiation and fungal opportunistic infections. (A) Time in months between ART initiation and histoplasmosis or cryptococcosis diagnosis (*n* = 50). Analysis by immunological status. (B) Time in months between ART initiation and histoplasmosis diagnosis (*n* = 36). Analysis by immunological status. (c) Time in months between ART initiation and cryptococcosis diagnosis (*n* = 16). Analysis by immunological status

Antiretroviral therapy (ART) initiation date was available on 50 out of 160 patients (31%). Among the 50 cases, 22 (44%) patients reported ART initiation more than 6 months prior to fungal OI diagnosis, and 28 (56%) patients reported starting ART less than 6 months prior to fungal OI diagnosis. From the 22 patients who reported ART initiation more than 6 months prior to fungal OI diagnosis, eight (36%) patients had CD4 cell counts >200 cells per µl and 14 (64%) patients had CD4 cell counts <200 cells per µl. However, from the 28 patients who reported starting ART between time of fungal OI diagnosis and 6 months prior to fungal OI diagnosis, only one patient (4%) had CD4 cell counts >200 cells per µl, and 27 (96%) patients had CD4 cell counts <200 cells per µl (Table 1; Figure 2).

The 30‐day mortality follow‐up showed that patients who were tested by *Histoplasma* Ag, regardless of positive or negative results, 13% of patients died (12/90). Among patients not tested for *Histoplasma* Ag, the 30‐day mortality was 75% (6/8); therefore, a significant difference in mortality was observed between tested and non‐tested patients (*p* = <0.001). In the other hand, there were no differences in the 30‐day mortality between patients who were tested and not tested for *Cryptococcosis* Ag, 20% mortality in patients tested for *Cryptococcosis* regardless of positive or negative results and 19% mortality in non‐tested patients (*p* = 0.914).

A total of 117 (73%) of the 160 patients with opportunistic fungal infections received antifungal treatment. A total of 102 (87%) were survived at 30 days post‐diagnosis, on the other 43 cases that were not treated by antifungals, a total of 29 (67%) survived at 30 days post‐diagnose (*p* = 0.004). By infection, eighty (82%) of the 98 patients with histoplasmosis were treated with antifungal, 71 (89%) of these patients survived at 30 days of diagnosis, in contrast with the 18 patients without antifungal treatment, a total of nine (50%) of the 18 that did not receive antifungal therapy survived at 30 days after the diagnosis of histoplasmosis (*p* = <0.001). In patients with cryptococcosis (*n* = 64), a total of 39 (61%) patients received antifungal treatment, 33 (85%) of these patients that were treated survived at 30 days post‐diagnose, on the other hand, of the total 25 patients that did no received antifungal treatment, a total of 20 (80%) patients survived at 30 days post‐cryptococcosis diagnosis (*p* = 0.631).

## 4 DISCUSSION

This study describes the results of a laboratory surveillance system for the detection of histoplasmosis and cryptococcosis in PLHIV in Guatemala and El Salvador, countries located in Central America. It is important to note that 85% of the cases identified in this surveillance were diagnosed using rapid tests. This result is in accordance with a recent study done in Guatemala, where researchers were able to confirm the important role of rapid diagnostics assays, and also were able to evaluated the performance of these diagnostics for the detection of fungal OI.^16^ In addition, this study reported the first and largest numbers of histoplasmosis and cryptococcosis cases from El Salvador.

Almost a half of the patients (75/160) in our cohort had been diagnosed with HIV within 3 months of the diagnosis of fungal OI, indicating that many patients in this population were seeking care in late stages of the disease when they were most vulnerable to fungal OIs. Only 33% (50/160) of those diagnosed with fungal OI were on ART at the time of fungal OI diagnosis, 56% (28/50) of those on ART had only started ART <6 months of fungal disease diagnosis and 44% (22/50) of those on ART reported starting ART more than 6 months prior to fungal OI diagnosis. The high number of patients diagnosed with fungal OI who reported starting ART more than 6 months ago (22/50), suggest interruptions in care and treatment, ART failure or poor adherence that was not reported.

Mortality was much lower than shown in a previous study for histoplasmosis done in Guatemala (18% vs 44%; *p* = 0.035).^10^ The decrease in mortality due to histoplasmosis was observed since the *Histoplasma* Ag testing started being used to diagnose histoplasmosis in 2009, due to rapid detection and early treatment of histoplasmosis.^7^, ^10^ In addition, in this study, we observed that 30‐day mortality was significantly lower in patients who were tested for *Histoplasma* Ag than patients who were not tested for *Histoplasma* Ag. Previous data about cryptococcosis mortality is lacking in these countries.^7^ There was no significant difference in the 30‐day mortality between tested and non‐tested patients for *Cryptococcus* Ag.

It was noted that antifungal treatment increased by 20% the probability of patients’ 30 days survival, especially in patients with histoplasmosis (39% increase). There were no significant differences in patients’ outcomes with cryptococcosis. Given the retrospective nature of this study and limited access to additional information, it was not possible to identify other factors involved in patients’ outcomes.

Co‐infections play a significant role in PLHIV, 44% of patients in this study were diagnosed with other OI. Due to the level of immunosuppression in these patients, the risk for developing fungal OI is high.^17^ The percentage of HIV patients who presented co‐infections in this study was similar to another's reports in Panama (25%), French Guiana (37%–42%), Argentina (42%), Brazil (43%) and Colombia (51%). In addition, TB was the most frequently reported OI in all these countries.^18^, ^19^, ^20^, ^21^, ^22^, ^23^ Risk factors and clinical characteristics of patients diagnosed with histoplasmosis and cryptococcosis were not different than reports in previous studies.^1^, ^2^

There were limitations to this study such as the lack of having the population denominator and the lack of screening asymptomatic PLHIV. The aim of this study was to describe the results of a laboratory‐based surveillance system for histoplasmosis and cryptococcosis, as such, all samples were from symptomatic PLHIV. This study was implemented in three hospitals, a follow‐up study including more hospitals and a larger population would be recommended in order to collect more data.

Reports of PLHIV with histoplasmosis and cryptococcosis are limited in Central America. International agencies and local Ministries of Health may consider continued support for reference labs in four major areas within the laboratory‐based surveillance of fungal OI: (1) strengthening regional laboratories and training laboratory staff in rapid test use and laboratory quality management; (2) providing training to Ministry of Health clinical staff in diagnosis of fungal OI; (3) strengthening health information systems; and (4) improving surveillance of fungal OI in Ministry of Health surveillance units.

Supporting clinicians to implement PEPFAR‐supported strategies that benefit PLHIV, such as rapid ART initiation, advanced HIV disease support, tracking and tracing of patients lost to follow‐up and treatment adherence support, are crucial to decrease fungal OIs among this population. Adequate and early fungal OI treatment is also important to lower the morbidity and mortality of PLHIV. The use of fungal screening, using rapid and highly sensitive and specific tests, and prophylactic fungal treatment among PLHIV are important preventive actions for HIV fungal co‐infections. Finally, strengthening HIV testing and counselling, as well as other HIV prevention strategies, is needed in Central American countries for earlier detection of at‐risk patients.

## CONFLICT OF INTEREST

No conflict of interest. The findings and conclusions in this report are those of the authors and do not necessarily represent the official position of the funding agencies.

## AUTHOR CONTRIBUTIONS

**Diana Forno:** Conceptualization (equal); Data curation (equal); Formal analysis (equal); Funding acquisition (equal); Investigation (equal); Methodology (equal); Project administration (equal); Resources (equal); Software (equal); Supervision (equal); Validation (equal); Visualization (equal); Writing‐original draft (equal); Writing‐review & editing (equal). **Blanca Samayoa:** Formal analysis (equal); Investigation (equal); Validation (equal); Writing‐original draft (equal); Writing‐review & editing (equal). **Narda Medina:** Investigation (equal); Writing‐original draft (equal); Writing‐review & editing (equal). **Eduardo Arathoon:** Investigation (equal); Methodology (equal); Validation (equal); Writing‐original draft (equal); Writing‐review & editing (equal). **Carlos Rodolfo Mejia:** Investigation (equal); Writing‐original draft (equal); Writing‐review & editing (equal). **Remei Gordillo:** Investigation (equal); Writing‐original draft (equal); Writing‐review & editing (equal). **Rolando Cedillos:** Investigation (equal); Writing‐original draft (equal); Writing‐review & editing (equal). **Jose Rodas:** Investigation (equal); Writing‐original draft (equal); Writing‐review & editing (equal). **Angela Ahlquist Cleveland:** Formal analysis (equal); Investigation (equal); Writing‐original draft (equal); Writing‐review & editing (equal). **Tom Chiller:** Conceptualization (equal); Funding acquisition (equal); Investigation (equal); Methodology (equal); Supervision (equal); Writing‐original draft (equal); Writing‐review & editing (equal). **Diego H. Caceres:** Data curation (equal); Formal analysis (equal); Investigation (equal); Methodology (equal); Project administration (equal); Resources (equal); Software (equal); Supervision (equal); Validation (equal); Visualization (equal); Writing‐original draft (equal); Writing‐review & editing (equal).

## Data Availability

The data that support the findings of this study are available from the corresponding author upon reasonable request.
